# *ANKK1* and *TH* gene variants in combination with paternal maltreatment increase susceptibility to both cognitive and attentive impulsivity

**DOI:** 10.3389/fpsyt.2022.868804

**Published:** 2022-07-22

**Authors:** Sara Palumbo, Veronica Mariotti, Stefano Vellucci, Klizia Antonelli, Nathaniel Anderson, Carla Harenski, Pietro Pietrini, Kent A. Kiehl, Silvia Pellegrini

**Affiliations:** ^1^Department of Surgical, Medical and Molecular Pathology and Critical Care, University of Pisa, Pisa, Italy; ^2^Department of Clinical and Experimental Medicine, University of Pisa, Pisa, Italy; ^3^The Mind Research Network and Lovelace Biomedical and Environmental Research Institute, Albuquerque, NM, United States; ^4^Molecular Mind Lab, IMT School for Advanced Studies Lucca, Lucca, Italy; ^5^Department of Psychology, University of New Mexico, Albuquerque, NM, United States

**Keywords:** cognitive/attentive impulsivity, parenting, gene variants, dopamine, *ANKK1*, rs1800497, *TH*, rs6356

## Abstract

Recent scientific findings suggest that dopamine exerts a central role on impulsivity, as well as that aversive life experiences may promote the high levels of impulsivity that often underlie violent behavior. To deepen our understanding of the complex gene by environment interplay on impulsive behavior, we genotyped six dopaminergic allelic variants (*ANKK1*-rs1800497, *TH*-rs6356, *DRD4*-rs1800955, *DRD4*-exonIII-VNTR, *SLC6A3*-VNTR and *COMT*-rs4680) in 655 US White male inmates convicted for violent crimes, whose impulsivity was assessed by BIS-11 (Barratt Impulsiveness Scale). Furthermore, in a subsample of 216 inmates from the whole group, we also explored the potential interplay between the genotyped dopaminergic variants and parental maltreatment measured by MOPS (Measure of Parental Style) in promoting impulsivity. We found a significant interaction among paternal MOPS scores, *ANKK1*-rs1800497-T allele and *TH*-rs6356-A allele, which increased the variance of BIS-11 cognitive/attentive scores explained by paternal maltreatment from 1.8 up to 20.5%. No direct association between any of the individual genetic variants and impulsivity was observed. Our data suggest that paternal maltreatment increases the risk of attentive/cognitive impulsivity and that this risk is higher in carriers of specific dopaminergic alleles that potentiate the dopaminergic neurotransmission. These findings add further evidence to the mutual role that genetics and early environmental factors exert in modulating human behavior and highlight the importance of childhood care interventions.

## Introduction

Impulsivity is the tendency to engage in fast and unplanned actions in response to either internal or external stimuli, with scarce or null consideration of consequences for themselves or other people (1).

Although impulsivity has developed as a spontaneous behavior with adaptive purposes that allow quick decisions when a fast response is required, high levels of impulsivity are usually maladaptive as they may produce bad conducts (2). High impulsivity, for example, underlies sensation/novelty seeking personalities (3), rule-breaking behaviors (e.g., risky driving, abnormal drug and alcohol consumption, excessive food intake, risky sexual behavior, gambling) (4–7), and mental disorders characterized by poor behavioral control, including attention deficit hyperactivity disorder, conduct disorder, substance abuse, bipolar disorder, borderline personality disorder and antisocial personality disorder (1, 8–10). Abnormal impulsivity may also promote antisocial behaviors, like vandalism, theft, aggression (11–15), and violence (16). Therefore, impulsivity is generally assessed in criminals [see, for instance, (17–20)] in order to provide them with an adequate forensic evaluation as well as suitable treatment planning and management (17). Violent offenders usually obtain higher impulsivity scores as compared to subjects convicted for non-violent crimes (21); additionally, greater impulsivity is often associated with higher rates of recidivism (22–26).

The dopaminergic system is thought to play a central role in impulsivity through the regulation of neural activity within the ventral and dorsal striatum (27–35). A reduced availability of dopamine receptor 2/3 (DRD_2/3_) in ventral striatum, for example, is associated with a greater impulsive behavior, both in rats (27–29) and in humans (30, 31). Moreover, genetic polymorphisms associated with an increased release of striatal dopamine or with a diminished availability of DRD_2_ and DRD_4_, predicted reward-related ventral striatum reactivity (32, 33). As known, ventral striatum is activated by novel and salient stimuli, thus playing a major role in processing appetitive/reward responses [see (36) for a review]. The dorsal striatum, instead, is involved in motor inhibitory control through the integration of sensorimotor, cognitive and motivational/emotional information (37, 38); a lower availability of DRD_2/3_ in dorsal striatum has been reported to predict impaired motor response inhibition in humans (34, 35).

Because of the role of dopaminergic system in impulsivity, we questioned whether distinct alleles of genes that modulate dopaminergic neurotransmission may affect impulsivity and criminal behavior. To this aim, we genotyped six allelic variants of the dopaminergic pathway (*ANKK1*-rs1800497, *TH*-rs6356, *DRD4*-rs1800955, *DRD4*-exonIII-VNTR, *SLC6A3*-VNTR and COMT-rs4680), in a large sample of adult violent male offenders, assessed for impulsivity by the Barratt Impulsiveness Scale, BIS-11 (39). Furthermore, as impulsivity traits appear to mediate the link between child maltreatment and adult criminal behavior (40) and specific dopaminergic genetic profiles have been reported to modulate the effect of child maltreatment on impulsive behavior (41), in a subsample of the same group of criminals, we also investigated the interaction between dopaminergic alleles and the effect of parental behavior on impulsivity.

## Materials and methods

The sample enrolled for this study included 655 US White male inmates convicted for violent crimes (age range: 18–65 years; mean age: 34.5 ± 10.6 years), belonging to two ethnicities: Latin/Hispanic (*n* = 375) and not-Latin/Hispanic (*n* = 298). The intelligence quotient (IQ) of these subjects was estimated by using the Wechsler Adult Intelligence Scale (42) (mean IQ: 97.44 ± 13.45).

Research was carried out in compliance with ethical standards and in accordance with the International Ethical Guidelines of the Declaration of Helsinki. Data were collected over more than a decade. The governing Institutional Review Board (IRB) is the Ethical and Independent Review Services (E&I); earlier versions included approval from the University of New Mexico Health Science Center IRB. Each participant provided a written informed consent to participate to the study. Subjects could withdraw from the study at any time.

Participants completed the self-report Barratt Impulsiveness Scale Version 11 (BIS-11) questionnaire (39, 43). Responses to each of the 30 BIS-11 items are rated on a 4-point Likert scale according to which statement better describes the individual behavior (1 = rarely/never; 2 = occasionally; 3 = often; 4 = almost always/always); thus, the total score ranges from a minimum of 30 to a maximum of 120 (BIS-11 Total score). Moreover, the scores for the three following domains were separately computed: (1) Cognitive/Attentional (BIS-11 Factor 1 score), which measures the difficulty to focus and pay attention on a task; (2) Motor (BIS-11 Factor 2 score), which measures the tendency to act rashly without any forethought; (3) Non-planning Impulsiveness (BIS-11 Factor 3 score), which measures the inability to adequately plan. Mean BIS-11 descriptive data are reported in Supplementary Table 1.

In a subsample of 216 criminals from the whole group, data about childhood environment were collected by the Measure of Parental Style (MOPS) instrument, a validated self-report questionnaire that measures the perceived parental indifference (e.g., uncaring, uninterested parents that leave children alone and forget about them), overcontrol (e.g., overprotective, overcontrolling and critical parents), and abuse (e.g., verbally and physically abusive parents who make their own children feel unsafe and in danger) experienced during the first 16 years of life (44). The MOPS questionnaire includes 15 items concerning the behavior of both parents. Participants rated each item as 0 (not true at all), 1 (slightly true), 2 (moderately true) or 3 (extremely true), as described in Parker et al. (44). A total score, ranging from 0 to 45, was calculated for each parent (Maternal MOPS score, *n* = 211; Paternal MOPS score, *n* = 185. There was no score if the parent had not been in the child’s life). Moreover, mother’s and father’s scores were summed to calculate a total score (MOPS Total score, *n* = 180). MOPS descriptive data are reported in Supplementary Table 2.

Each participant also provided a sample of saliva for DNA analysis by an Oragene collection tube (DNA Genotek Inc., Kanata, Ontario, Canada). DNA was extracted from saliva by the prepITL2P kit according to the manufacturer’s protocol (DNA Genotek Inc., Kanata, ON, Canada). Six candidate genetic variants of the dopaminergic pathway were genotyped: *ANKK1*-rs1800497, *TH*-rs6356, *DRD4*-rs1800955, *DRD4*-exonIII-VNTR, *SLC6A3*-VNTR and *COMT*-rs4680. The following pairs of primers were designed by using the Beacon Designer v.8 software (PREMIER 128 Biosoft, Palo Alto, CA, United States) and used for Polymerase Chain Reaction (PCR) amplification:

– 5′-TGCAGCTCACTCCATCCTG-3′ and 5′-GCAACA CAGCCATCCTCAAA-3′ for *ANKK1*-rs1800497;
– 5′-CTTTGAGGAGAAGGAGGGGA-3′ and 5′-ACC TCAAACACCTTCACAGC-3′ for *TH*-rs6356;
– 5′-GGATGAGCTAGGCGTCGG-3′ and 5′-CTCACC CTAGTCCACCTGG-3′ for *DRD*_4_-rs1800955;
– 5′-GCGACTACGTGGTCTACTCG-3′ and 5′-AGGAC CCTCATGGCCTTG-3′ for *DRD4*-exonIII-VNTR;
– 5′-TGTGGTGTAGGGAACGGCCTGAG-3′ and
– 5′-CTTCCTGGAGGTCACGGCTCAAGG-3′ for *SLC6A3*-VNTR;
– 5′-CAGCGGATGGTGGATTTC-3′ and 5′-TTCCAGG TCTGACAACGG-3′ for *COMT*-rs4680.

*DRD4*-exonIII-VNTR and *SLC6A3*-VNTR were genotyped by running the PCR amplicons on 2% ethidium bromide-stained agarose gel. *DRD4*-rs1800955 was genotyped by PCR-Restriction Fragment Length Polymorphism (RFLP) by using the restriction endonuclease *Fsp*I (New England BioLabs Inc., Ipswich, MA, United States). *ANKK1*-rs1800497, *TH*-rs6356, and C*OMT*-rs4680 were genotyped by High Resolution Melting (HRM)-PCR using the CFX Connect instrument (Bio-Rad, Hercules, CA, United States) and the Bio-Rad Precision Melt Analysis software.

The Hardy–Weinberg equilibrium (χ^2^ test) was assessed for each allelic variant.

As far as Single Nucleotide Polymorphisms are concerned, homozygotes for the minor allele were grouped with heterozygotes (i.e., *ANKK1*-rs1800497 T/T + C/T, *TH*-rs6356 A/A + A/G, *DRD4*-rs1800955 C/C + C/T, *COMT*-rs4680 A/A + A/G) and compared to the homozygotes for the ancestral allele.

For the VNTR (variable number tandem repeats) polymorphisms, genotypes were grouped based on their functional effect, as reported in the scientific literature. Specifically, the *DRD4*-exonIII-VNTR low activity allele (7r) was compared to the high activity alleles (non-7r) (45–50), while the *SLC6A3*-VNTR low activity genotype (10r/10r) was compared to the high activity allele (9r) (32, 33, 51–53).

Statistical analysis was performed by using the SPSS 21 software package (IBM Corporation, Armonk, NY, United States). For each variable, the deviation from a normal distribution was assessed by the Shapiro-Wilk test. Outliers were searched by the Interquartile Range (IQR) method [previously described in Jones PR (54)].

Generalized estimating equations (GEE), with an exchangeable working matrix and Tweedie model with identity link function, were used to model the associations between BIS-11 and MOPS scores, between genotype and BIS-11 scores, and among genotype, BIS-11 and MOPS scores.

Age significantly correlated with BIS-11 Total (ρ_s_ = −0.142, *p* = 2.6 × 10^–4^), Factor 1 (ρ_s_ = −0.166, *p* = 9 × 10^–6^), and Factor 2 (ρ_s_ = −0.87, p_Bonf._ = 0.042) scores, but not with Factor 3 (ρ_s_ = −0.78, p_Bonf._ = 0.087) scores.

IQ significantly correlated with BIS-11 Total (ρ_s_ = −0.152, *p* = 1.04 × 10^–4^), Factor 1 (ρ_s_ = −0.113, p_Bonf._ = 0.006), and Factor 3 (ρ_s_ = −0.151, p_Bonf._ = 6.9 × 10^–5^) scores, but not with Factor 2 scores (ρ_s_ = −0.058, p_Bonf._ = 0.315).

Between the two ethnic groups (Latin/Hispanic and not-Latin/Hispanic), there were no significant differences in BIS-11 scores (Total scores: Mann–Whitney *U* test z-score = −1.484, *p* = 0.414; Factor 1 scores: Mann–Whitney *U* test z-score = −0.068, p_Bonf._ = 1; Factor 2 scores: Mann–Whitney *U* test z-score = −1.511, p_Bonf._ = 0.131; Factor 3 scores: Mann–Whitney *U* test z-score = −0.160, p_Bonf._ = 1), but there was a significant weak influence on MOPS Total scores (Mann–Whitney *U* test z-score = 2.083, *p* = 0.037). No differences emerged, instead, neither in Maternal (Mann–Whitney *U* test z-score = 2.073, p_Bonf._ = 0.076) nor in Paternal MOPS (Mann–Whitney *U* test z-score = 1.747, p_Bonf._ = 0.162) scores.

Finally, the distribution of genotype groupings differed between Latin/Hispanic and not-Latin/Hispanic subjects for *ANKK1*-rs1800497 (Fisher’s exact test: p_Bonf._ = 0.006) and *COMT*-rs4680 (Fisher’s exact test: p_Bonf._ = 0.008), but not for *TH*-rs6356 (Fisher’s exact test: p_Bonf._ = 0.119), *DRD4*-rs1800955 (Fisher’s exact test: p_Bonf._ = 1), *DRD4*-exonIII-VNTR (Fisher’s exact test: p_Bonf._ = 0.165) and SLC6A3-VNTR (Fisher’s exact test: p_Bonf._ = 1).

Association analyses were, therefore, adjusted for age, IQ, and ethnicity.

In addition, a linear regression analysis allowed for the prediction of the amount of variance of BIS-11 scores explained by MOPS scores *per se* or in interaction with genotype. A stepwise linear regression was used to predict the contribution of both Paternal and Maternal MOPS scores to the variance of BIS-11 scores.

Significance level was set according to the Bonferroni method, considering the number of simultaneously tested hypotheses.

## Results

Forty-one percent (*n* = 267) of the enrolled criminals had abnormally elevated scores of impulsivity (BIS-11 scores ≥ 72) (55).

The distribution of genotype frequencies for all the six polymorphisms was in Hardy–Weinberg equilibrium both in the whole sample (*n* = 655) with BIS-11 data only and in the subsample of subjects (*n* = 216) with both BIS-11 and MOPS data (Table 1).

**TABLE 1:** Genotype frequencies and Hardy–Weinberg equilibrium (χ^2^ test).

		Whole sample of 655 criminals			Subsample of 216 criminals with both BIS-11 and MOPS data		
		
Polymorphism	Genotype	*N*	Frequency	Hardy–Weinberg equilibrium	*N*	Frequency	Hardy–Weinberg equilibrium
*ANKK1*-rs1800497	T/T	55	0.084	χ^2^ = 0.353	16	0.074	χ^2^ = 1.122
	C/T	258	0.396	*p* = 0.552	97	0.449	*p* = 0.290
	C/C	339	0.520		103	0.477	
*TH*-rs6356	A/A	106	0.164	χ^2^ = 0.230	37	0.172	χ^2^ = 0.026
	A/G	319	0.493	*p* = 0.632	102	0.479	*p* = 0.872
	G/G	222	0.343		75	0.349	
*DRD4*-rs1800955	C/C	122	0.189	χ^2^ = 0.300	35	0.165	χ^2^ = 0.007
	C/T	310	0.479	*p* = 0.584	102	0.486	*p* = 0.933
	T/T	215	0.332		74	0.349	
*DRD4*-exonIII-VNTR	7r/7r	36	0.055	χ^2^ = 1.811	6	0.028	χ^2^ = 0.482
	7r/non-7r	208	0.321	*p* = 0.178	68	0.321	*p* = 0.488
	non-7r/non-7r	405	0.624		137	0.651	
*SLC6A3*-VNTR	9/9	27	0.045	χ^2^ = 2.061	12	0.057	χ^2^ = 0.245
	9/10	237	0.374	*p* = 0.151	71	0.338	*p* = 0.621
	10/10	370	0.584		126	0.605	
*COMT*-rs4680	A/A	140	0.219	χ^2^ = 0.463	43	0.204	χ^2^ = 0.035
	A/G	309	0.484	*p* = 0.496	102	0.488	*p* = 0.851
	G/G	190	0.297		65	0.308	

Data are reported for the whole sample of 655 criminals with only BIS-11 data and for the subsample of 216 criminals with both BIS-11 and MOPS data. r = repeats.

None of the analyzed polymorphisms showed any statistically significant association with BIS-11 scores (Supplementary Table 3).

Mean Maternal MOPS scores (8.73 ± 7.88) were significantly lower than mean Paternal MOPS scores (12.30 ± 11.09; Wilcoxon signed-rank test: Z = 4.387, *p* < 10^–4^; Supplementary Table 2).

### Correlations between Barratt Impulsiveness Scale and Measure of Parental Style questionnaire

Measure of Parental Style total scores were not significantly correlated to BIS-11 Total scores (Waldχ^2^ = 2.847, df = 1, *p* = 0.092).

**FIGURE 1:**
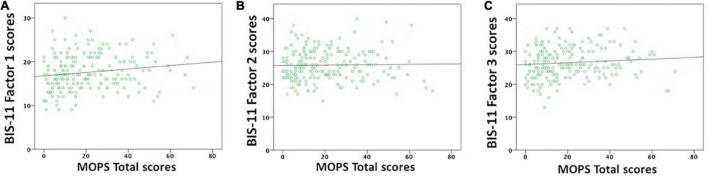
Correlations between BIS-11 subscales and MOPS Total scores. MOPS Total scores significantly correlated with BIS-11 **(A)** cognitive/attentive (Factor 1) scores, but not with **(B)** motor (Factor 2) and **(C)** non-planning (Factor 3) scores.

Concerning BIS-11 subscales, MOPS Total scores positively correlated with BIS-11 Factor 1 scores (Waldχ^2^ = 6.153, df = 1, p_Bonf._ = 0.039; Figure 1A), but not with Factor 2 (Waldχ^2^ = 0.029, df = 1, p_Bonf._ = 1; Figure 1B) and Factor 3 (Waldχ^2^ = 1.769, df = 1, p_Bonf._ = 0.549; Figure 1C) scores.

**FIGURE 2:**
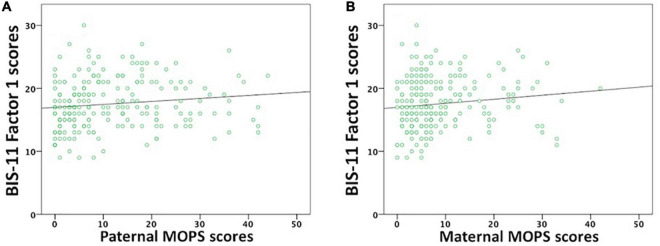
Correlations between BIS-11 cognitive/attentive (Factor 1) and both Paternal and Maternal MOPS scores. BIS-11 cognitive/attentive scores significantly correlated with **(A)** Paternal MOPS scores, but not with **(B)** Maternal MOPS scores.

BIS-11 Factor 1 scores positively correlated with Paternal MOPS scores (Waldχ^2^ = 6.153, df = 1, p_Bonf._ = 0.039; Figure 2A) but not with Maternal MOPS scores (Waldχ^2^ = 2.707, df = 1, p_Bonf._ = 0.3; Figure 2B).

Indeed, a stepwise linear regression analysis showed that Paternal MOPS scores produced a significant model that explained 1.8% (*R*^2^ = 0.024, *F*_1,178_ = 4.332, *p* = 0.039; β = 0.154) of the variance of BIS-11 Factor 1 scores, while Maternal MOPS scores did not contribute to this variance.

### Genotype by Paternal MOPS by BIS-11 Factor 1 score interaction

**FIGURE 3:**
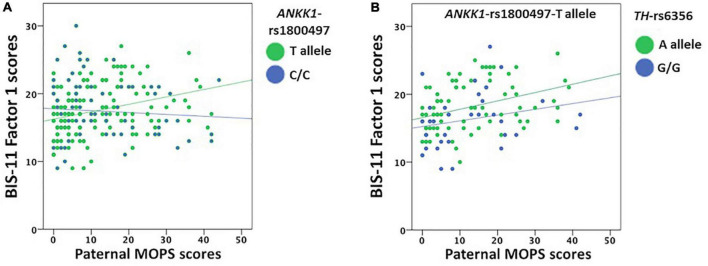
Correlation between BIS-11 cognitive/attentive (Factor 1) and Paternal MOPS scores divided by *ANKK1*-rs1800497 genotype groupings and in *ANKK1*-rs1800497-T allele carriers divided by *TH*-6356 genotype groupings. **(A)** In *ANKK1*-rs1800497-T allele carriers, Paternal MOPS scores positively correlated with BIS-11 cognitive/attentive scores. **(B)** Among *ANKK1*-rs1800497-T allele carriers, Paternal MOPS scores positively correlated with BIS-11 cognitive/attentive scores in *TH*-rs6356-A allele carriers.

After including genetics in the analysis, *ANKK1*-rs1800497 significantly influenced the correlation between Paternal MOPS scores and BIS-11 Factor 1 scores (Waldχ^2^ = 13.178, df = 2, p_Bonf._ = 0.006). Specifically, Paternal MOPS scores positively correlated with BIS-11 Factor 1 scores in *ANKK1*-rs1800497-T allele carriers (p_Bonf._ = 0.002; Figure 3A), but not in *ANKK1*-rs1800497-C/C genotype carriers (p_Bonf._ = 1).

The interaction between Paternal MOPS scores and *ANKK1*-rs1800497-T allele increased the explained variance of BIS-11 Factor 1 scores up to 12.7% (R^2^ = 0.136, F_1,93_ = 14.62, p_Bonf._ < 10^–6^; β = 0.369), while the C/C genotype produced a non-significant model (p_Bonf._ = 1).

None of the other analyzed polymorphisms showed any significant direct effect on the correlation between Paternal MOPS and BIS-11 Factor 1 scores (Supplementary Table 4).

However, the *ANKK1*-rs1800497-T allele significantly interacted with *TH*-rs6356 (Waldχ^2^ = 26.351, df = 2, p_Bonf._ = 1 × 10^–5^). Indeed, among the *ANKK1*-rs1800497-T allele carriers, Paternal MOPS scores positively correlated with BIS-11 Factor 1 scores in the *TH*-rs6356-A allele carriers (p_Bonf._ < 10^–6^; Figure 3B), but not in the *TH*-rs6356-G/G genotype carriers (p_Bonf._ = 1). Moreover, in the *ANKK1*-rs1800497-T allele carriers, the interaction between Paternal MOPS scores and *TH*-rs6356-A allele increased the variance of BIS-11 Factor 1 scores up to 20.5% (R^2^ = 0.217, F_1,66_ = 117.99, p_Bonf._ < 10^–6^; β = 0.466), while the *TH*-rs6356-G/G genotype produced a non-significant model (p_Bonf._ = 0.63).

None of the other polymorphisms significantly interacted with the *ANKK1*-rs1800497-T allele (Supplementary Table 5).

## Discussion

In the present study, we investigated the potential association between six candidate genetic variants of the dopaminergic pathway (*ANKK1*-rs1800497, *TH*-rs6356, *DRD4*-rs1800955, *DRD4*-exonIII-VNTR, *SLC6A3*-VNTR, *COMT*-rs4680) and impulsivity within a large sample of US male inmates convicted for violent crimes. In a subsample of the same group of criminals, we also examined the influence on impulsivity of the interaction between the dopaminergic genetic variants and the individual parental style experienced during the first 16 years of life.

Our data did not find a nominal influence on impulsivity scores by anyone of the analyzed polymorphisms taken individually. These results, although in contrast with other studies that showed a significant interaction between BIS-11 Total scores and *SLC6A3*-VNTR (32), *DRD4*-exonIII-VNTR (56) or *COMT*-rs4680 (56) and between BIS-11 non-planning scores and *COMT*-rs4680 (57), are in line with some previous findings showing no association between BIS-11 Total scores and *SLC6A3*-VNTR (58, 59), *TH*-rs6356 (60), *DRD4*-exonIII-VNTR (32), *COMT*-rs4680 (32, 58, 60), or *ANKK1*-rs1800497 (56, 60).

Of note, the data reported by Forbes (32) and Soeiro-De-Souza (57) would lack statistical significance if corrected for multiple comparisons, while the sample enrolled in the Varga’s study (56) included non-institutionalized subjects with mixed gender and different age, ancestry and ethnicity as compared to our sample. This may contribute to explain why we did not observe the same associations (61, 62).

Concerning the role of the environment, we found that Paternal MOPS scores explained only 1.8% of the variance of the BIS-11 cognitive/attentive scores. As the cognitive/attentive subscale of BIS-11 evaluates the inability to concentrate and pay attention on current tasks and to focus thoughts (63), our data suggest that paternal indifference, over-control and abuse may exert a weak, yet significant, influence on the normal development of child cognitive skills, favoring more distracting and moving thoughts, as well as a restless behavior.

Maternal MOPS scores were not significantly associated with BIS-11 scores in our sample. However, as most of the criminals recruited in the present study reported low Maternal MOPS scores, thus suggesting that they had not been exposed to dysfunctional mothering, our data do not allow to exclude that a negative mother’s parenting may exert an impact on impulsivity as well.

In line with our results, several previous studies showed that early traumas, including childhood maltreatment, may affect impulsivity (64, 65) and the executive functions impaired in cognitive/attentive impulsivity (66) (e.g., memory, attention, concentration, conceptualization, verbal comprehension, spatial orientation, and analysis and synthesis abilities) [see Su et al. (67) for a comprehensive review and Liu (68) for a meta-analysis]. None of the previous studies, however, focused on the distinct impact of mothering and fathering. Thus, our data indicate for the first time that paternal parenting influences the normal development of cognitive/attentive impulsivity, producing long-lasting behavioral consequences. This observation is in agreement with the United States 2006 Child Abuse and Neglect User Manual Series, which describes as involved fathers play a beneficial role on child verbal skill development, intellectual functioning, academic achievement, and cognitive capacity (69).

Our results also showed that the impact of paternal maltreatment on cognitive/attentive impulsivity is strengthened by the interaction with the *ANKK1*-rs1800497-T allele. The BIS-11 cognitive/attentional scores variance explained by Paternal MOPS scores, indeed, increased significantly from 1.8 to 12.7%.

Consistently, previous studies observed that the *ANKK1*-rs1800497-T allele interacts with both prenatal and rearing adverse environments, negatively affecting the offspring executive functions, and predisposing to irritability, attention deficits and violence (70–72). Moreover, female offenders carrying the *ANKK1*-rs1800497-T allele, born from criminal fathers, more often present persistent and violent delinquency (73), in line with the role of *ANKK1*-rs1800497-T allele in increasing vulnerability to maladaptive fathering. Of note, the *ANKK1*-rs1800497-T allele has been previously associated with attention deficit hyperactivity disorder (ADHD) (74–76) and with deficits in sustaining attention observed in individuals with alcohol dependence (74).

*ANKK1*-rs1800497 (C/T) is a missense variant producing a glutamine to lysine amino acidic change at position 713, located close to the dopamine receptor D2 (*DRD*_2_) gene. The *ANKK1*-rs1800497-T allele has been shown to decrease by 30–40% the DRD_2_ expression in the striatum (77–85). More specifically, the *ANKK1*-rs1800497-T allele seems to reduce the expression of the S isoform of DRD_2_. DRD_2_S mainly acts as a presynaptic inhibitory auto-receptor, which inhibits the dopamine release through negative feedback on dopaminergic neurons and promotes the dopamine reuptake by facilitating the expression of the dopamine transporter on the surface of presynaptic terminals (86, 87). DRD_2_S also acts as a heteroreceptor, by modulating the release of GABA, glutamate and acetylcholine from striatal interneurons (88, 89) and the glutamate inputs from sensory and motor cortical areas (89–91); DRD_2_S is abundantly expressed on striatal dopamine terminals (92) and seems to be the main regulator of striatal function (93).

Finally, the striatal DRD_2_S appears to be critically involved in the regulation of the default mode network (DMN) (93), a circuit that comprises several brain regions (from prefrontal cortex to medial posterior cortex to lateral areas, including inferior parietal lobule and medial temporal lobes) functionally connected to the striatum (94–96). The DMN acts as a sentinel of the surrounding environment that allows directing attention toward or away from external stimuli; the activity of DMN is maxima during rest but is reduced during attention-demanding and externally oriented tasks [for example, see Anticevic et al. (97)]. An altered DMN activity has been associated with attention deficits; patients with ADHD, for example, show difficulties in suppressing DMN activity on attention-demanding tasks (98, 99), as well as subjects with high BIS-11 cognitive/attentive scores show a higher connectivity within the DMN (100).

As early life traumas have been demonstrated to modify the functional connectivity between the DMN and the striatum (101, 102) and to alter the striatal dopamine turnover (103), the impact of traumas on the DMN connectivity might be mediated by their effects on dopamine signaling. We thus hypothesize that the *ANKK1*-rs1800497-T allele and paternal maltreatment may synergistically hamper the disengagement of DMN, necessary in goal-directed and attention-demanding tasks (104), thus favoring the attentive/cognitive impulsivity.

Finally, we observed that the effect of *ANKK1*-rs1800497-T allele in interaction with paternal maltreatment on cognitive/attentive impulsivity was further increased by the presence of the *TH*-rs6356-A allele. More specifically, the variance of BIS-11 cognitive/attentive scores, explained by Paternal MOPS scores, increased up to 20.5% in carriers of both *ANKK1*-rs1800497-T and *TH*-rs6356-A alleles.

The *TH*-rs6356 (G/A) is a missense variant of the gene coding for the tyrosine hydroxylase (TH), the enzyme deputed to the hydroxylation of the amino acid L-tyrosine into L-3, 4-dihydroxyphenylalanine (L-DOPA), a rate-limiting step for dopamine synthesis (105).

As DRD_2_S has been shown to deactivate the catalytic activity of TH in the striatum by inhibiting the enzyme phosphorylation (106), we hypothesize that the reduced expression of DRD_2_S mediated by the *ANKK1*-rs1800497-T allele may result in a higher TH phosphorylation that produces an increased dopamine synthesis. Concerning the *TH*-rs6356-A allele, its function is not known yet, while it is known its location in a regulatory domain of *TH.* This suggests that it might affect the TH catalytic activity and likely cooperate with the *ANKK1*-rs1800497-T allele in increasing the dopamine synthesis rate.

Overall, these findings deepen our understanding of the complex interplay between nature and nurture in the modulation of impulsive behavior. Specifically, in a large sample of criminals, we detected a synergistic interaction in promoting attentive/cognitive impulsivity between two dopaminergic genetic variants that cooperate in increasing dopaminergic neurotransmission and paternal maladaptive parenting. More in general, these results highlight the reciprocally connected role of genetics on one hand and of early life environment and childhood parental care on the other hand in shaping the individual ability to modulate their behavior in adult life.

## Data availability statement

The original contributions presented in this study are included in the article/Supplementary Material, further inquiries can be directed to the corresponding author.

## Ethics statement

The studies involving human participants were reviewed and approved by governing Institutional Review Board (IRB) and the University of New Mexico Health Science Center IRB. The patients/participants provided their written informed consent to participate in this study.

## Author contributions

SPe, PP, and KK conceived and supervised the study. SPe acquired the project funding. CH, NA, and KK recruited the sample and collected psychometric data and salivas. CH and NA organized the sample database. SPa and SPe planned the experimental design, interpreted the results, and wrote the manuscript. SPa, VM, KA, and SV carried out the experiments. SPa performed the statistical analysis and produced figures and tables. VM contributed to interpret the results and write the manuscript. PP and KK critically reviewed the manuscript. All authors edited and approved the final version of the manuscript.

## Conflict of interest

The authors declare that the research was conducted in the absence of any commercial or financial relationships that could be construed as a potential conflict of interest.

## Publisher’s note

All claims expressed in this article are solely those of the authors and do not necessarily represent those of their affiliated organizations, or those of the publisher, the editors and the reviewers. Any product that may be evaluated in this article, or claim that may be made by its manufacturer, is not guaranteed or endorsed by the publisher.
