# Supplementary material for: ANKK1 and TH gene variants in combination with paternal maltreatment increase susceptibility to both cognitive and attentive impulsivity
Source: Front Psychiatry. 2022 Jul 22;13:868804. doi: 10.3389/fpsyt.2022.868804 (PMC9352854; doi:10.3389/fpsyt.2022.868804)
Supplement: Supplementary file 1 [file Data_Sheet_1.PDF]

Supplementary Tables

Supplementary Table 1. BIS-11 descriptive data. Data are reported for the whole sample of 655 criminals with only BIS-11 data and for the subsample of 216 criminals with both BIS-11 and MOPS data. MIN= minimum, MAX= maximum, SD= standard deviation.

	**Whole sample of 655 criminals**				**Subsample of 216 criminals**			
	**min**	**max**	**mean**	**SD**	**min**	**max**	**mean**	**SD**
**Factor 1**	8	30	17.62	4.063	9	30	17.46	3.914
**Factor 2**	14	40	25.36	4.721	15	40	25.67	4.645
**Factor 3**	12	43	26.78	5.123	13	38	26.38	4.800
**TOTAL**	42	106	69.76	11.385	45	97	69.51	10.520

Supplementary Table 2. MOPS descriptive data and Wilcoxon signed-rank test between mean Maternal MOPS scores and mean Paternal MOPS scores for the subsample of 216 criminals. MIN= minimum, MAX= maximum, SD= standard deviation.

	Sample size	min	max	Mean ± SD	Wilcoxon signed-rank test
Maternal MOPS	211	0	42	8.73 ± 7.88	Z= 4.387, $p < 10^{-4}$
Paternal MOPS	185	0	44	12.30 ± 11.09	

Supplementary Table 3. Association between each polymorphism and BIS-11 Total, cognitive/attentive (Factor1), motor (Factor 2) and non-planning (Factor 3) scores, before and after Bonferroni correction.

	Wald Chi-square	df	p value	p_{Bonf}
***ANKK1*-rs1800497 * BIS-11 Total**	0.028	1	0.866	1
***ANKK1*-rs1800497 * BIS-11 Factor 1**	0.007	1	0.934	1
***ANKK1*-rs1800497 * BIS-11 Factor 2**	0.711	1	0.399	1
***ANKK1*-rs1800497 * BIS-11 Factor 3**	0.091	1	0.763	1
***TH*-rs6356 * BIS-11 Total**	1.075	1	0.3	1
***TH*-rs6356 * BIS-11 Factor 1**	0.213	1	0.645	1
***TH*-rs6356 * BIS-11 Factor 2**	2.105	1	0.147	1
***TH*-rs6356 * BIS-11 Factor 3**	0.359	1	0.549	1
***DRD4*-rs1800955 * BIS-11 Total**	0.757	1	0.384	1
***DRD4*-rs1800955 * BIS-11 Factor 1**	0	1	0.994	1
***DRD4*-rs1800955 * BIS-11 Factor 2**	2.062	1	0.151	1
***DRD4*-rs1800955 * BIS-11 Factor 3**	0.391	1	0.532	1
***DRD4*-exonIII-VNTR * BIS-11 Total**	3.125	1	0.077	0.462
***DRD4*-exonIII-VNTR * BIS-11 Factor 1**	1.098	1	0.295	1
***DRD4*-exonIII-VNTR * BIS-11 Factor 2**	3.677	1	0.055	0.99
***DRD4*-exonIII-VNTR * BIS-11 Factor 3**	1.702	1	0.192	1
***SLC6A3*-VNTR * BIS-11 Total**	0.353	1	0.553	1
***SLC6A3*-VNTR * BIS-11 Factor 1**	0.384	1	0.563	1
***SLC6A3*-VNTR * BIS-11 Factor 2**	2.18	1	0.14	1
***SLC6A3*-VNTR * BIS-11 Factor 3**	0.137	1	0.712	1
***COMT*-rs4680 * BIS-11 Total**	0.607	1	0.436	1
***COMT*-rs4680 * BIS-11 Factor 1**	0.153	1	0.695	1
***COMT*-rs4680 * BIS-11 Factor 2**	0.708	1	0.4	1
***COMT*-rs4680 * BIS-11 Factor 3**	1.704	1	0.192	1

Supplementary Table 4. Interaction among each SNP, Paternal MOPS scores and BIS cognitive/attentive scores, before and after Bonferroni correction.

	**Wald chi-square**	**df**	**p**	$\mathbf{p_{Bonf}}$
***ANKK1*-rs1800497**	13.178	2	0.001	0.006
***TH*-rs6356**	6.114	2	0.047	0.282
***DRD4*-rs1800955**	6.561	2	0.038	0.228
***DRD4*-exonIII-VNTR**	6.196	2	0.045	0.270
***SLC6A3*-VNTR**	6.4	2	0.041	0.246
***COMT*-rs4680**	6.845	2	0.033	0.198

Supplementary Table 5. In *ANKK1*-rs1800497-T allele carriers, interaction among each SNP, Paternal MOPS scores and BIS cognitive/attentive scores, before and after Bonferroni correction.

***ANKK1*-rs1800497-T allele carriers**	**Wald Chi-square**	**df**	**p**	**p_{Bonf}**	**Post hoc for genotype groupings**	**p value**	**p_{Bonf}**
***TH*-rs6356**	26.351	2	2×10^{-6}	1×10^{-5}	**A allele**	$<10^{-6}$	$<10^{-6}$
					**G/G**	0.138	1
***DRD4*-rs1800955**	19.106	2	7.1×10^{-5}	3.6×10^{-4}	**C allele**	$6.8\text{x}10^{-5}$	$6.8\text{x}10^{-4}$
					**T/T**	0.004	0.04
***DRD4*-exonIII-VNTR**	21.1	2	2.6×10^{-5}	1.3×10^{-4}	**non-7r**	$2\text{x}10^{-5}$	$2\text{x}10^{-4}$
					**7r allele**	0.004	0.04
***SLC6A3*-VNTR**	19.423	2	6.1×10^{-5}	3.1×10^{-4}	**non 9r**	$1.19\text{x}10^{-4}$	$1.19\text{x}10^{-3}$
					**9r allele**	0.005	0.05
***COMT*-rs4680**	18.826	2	8.2×10^{-5}	4.1×10^{-4}	**A allele**	$6.1\text{x}10^{-5}$	$6.1\text{x}10^{-4}$
					**G/G**	0.0007	0.007
